# ESSENTIALS OF DERMATOLOGY – FOR ALL SECTIONS OF MEDICAL FRATERNITY

**Published:** 2009

**Authors:** S R Sengupta

**Affiliations:** *Former Professor & Head, Department of Dermatology, IPGMER & SSKM Hospital, Kolkata, India*

The second edition of Essentials of Dermatology has come out with a new look and high standard of cover page, color illustrations and printing. Three new chapters on skin in systemic disease, skin changes of pregnancy and old age and anti retroviral therapy (ART) have been added giving a wider dimension to its contents. The existing chapters have also been revised and updated to keep pace with newer developments in dermatology.

This handbook has incorporated all basic principles of diagnosis of skin diseases and provided a brief update and essential information on disease processes. An algorithmic approach to diagnosis would have added color to it. This brief but essential information on the diseases and high standard color photographs will make dermatology more accessible to new entrants Post Graduate Trainees (PGTs), general physicians as well as senior dermatologists. It is worth carrying, preserving in any library and is also recommended for all sections of the medical fraternity.

One may have some reservations regarding selection criteria of most influential persons in Dermatology, Venereology and Leprosy in India but that does not necessarily undermine the quality of the book.

